# Plant sterols–enriched diet decreases small, dense LDL-cholesterol levels in children with hypercholesterolemia: a prospective study

**DOI:** 10.1186/1824-7288-40-42

**Published:** 2014-05-03

**Authors:** Anastasia Garoufi, Styliani Vorre, Alexandra Soldatou, Charalampos Tsentidis, Lydia Kossiva, Antonios Drakatos, Antonios Marmarinos, Dimitrios Gourgiotis

**Affiliations:** 1 Lipid Outpatient Clinic, Second Department of Pediatrics, Athens University, Athens, Greece; 2 Second Department of Pediatrics, Athens University, Athens, Greece; 3 Biochemistry Laboratory, “P. & A. Kyriakou” Children’s Hospital, Athens, Greece; 4 Research Laboratories, Second Department of Pediatrics, Athens University, Athens, Greece

**Keywords:** sdLDL, LDL-C, Dyslipidemia, Phytosterols

## Abstract

**Background:**

Small dense low density lipoprotein-cholesterol (sdLDL-C) molecules are more atherogenic compared with large buoyant ones. Phytosterols-enriched diets are effective in decreasing total cholesterol (TC) and low density lipoprotein-cholesterol (LDL-C) concentrations in hyperlipidemic children without significant adverse effects. Limited data on the impact of such a diet on sdLDL-C levels is available in adults while there are no reports concerning children. The purpose of this study is to prospectively evaluate the effect of the daily consumption of 2 g of plant sterols on sdLDL-C levels in children with hypercholesterolemia.

**Methods:**

Fifty-nine children, 25 with LDL-C ≥ 3.4 mmol/l (130 mg/dl) and 34 with LDL-C < 3.4 mmol/l, aged 4.5-15.9 years, were included in the study. A yogurt-drink enriched with 2 g of plant sterols was added to the daily diet of hypercholesterolemic children and 6–12 months later lipid profiles were reassessed. Direct quantitative methods were used to measure LDL-C and sdLDL-C levels.

**Results:**

The consumption of plant sterols reduced sdLDL-C significantly (p < 0.001), but levels remained higher compared with controls (p < 0.001). TC, LDL-C, non high density lipoprotein-cholesterol (NonHDL-C) and apolipoprotein B (ApoB) levels also decreased significantly (p < 0.05). The median reduction of sdLDL-C and LDL-C was 16.6% and 13%, respectively. These variables decreased >10% in sixteen children (64%), independently from baseline levels, sex, age and body mass index (BMI). High density lipoprotein-cholesterol (HDL-C), lipoprotein a [Lp(a)], and triglycerides (TGs) levels remained unaffected.

**Conclusions:**

Plant sterols decrease sdLDL-C significantly and may be beneficial for children with hypercholesterolemia.

## Background

Low density lipoprotein-cholesterol (LDL-C) comprises a heterogeneous group of particles that vary in size, density, lipid composition, electrical charge and functional properties. Two subtypes are recognized, small dense LDL (sdLDL), also known as phenotype B and the large, buoyant LDL (lbLDL) particles or phenotype A [1]. Genetic and non-genetic factors influence the distribution of LDL subclasses in the plasma [2,3]. Compared with lbLDL, sdLDL subclasses have been suggested to be more atherogenic as a result of their prolonged plasma half-life, lower binding affinity for LDL receptors, higher degree of penetration to the arterial wall, and lower resistance to oxidative stress [4]. An increased risk for coronary heart disease (CHD) in subjects with phenotype B has been previously documented [5,6].

Many studies in adults and children with dyslipidemia have shown that phytosterols-enriched diets decrease TC and LDL-C concentrations effectively, without any serious adverse effects; albeit with heterogeneity in their response [7]. Expert Panel on integrated guidelines for cardiovascular health and risk reduction in children and adolescents recommend the use of plant sterols/stanols, up to 2 g/day as a supportive action in children older than 2 years old with familial hypercholesterolemia (FH) [8]. In a recent report of the Maastricht meeting, based on the results of more than 200 clinical trials, the use of plant sterols is thought to be favorable although their potentially atherogenic action remains to be clarified [9].

The data regarding the effect of phytosterols supplements on the more atherogenic sdLDL particles is limited in adults, while lacking in children with hyperlipidemia.

In the present prospective study we investigated the changes in sdLDL-C levels following the daily consumption of 2 g of plant sterols in the form of a yogurt-drink in children and adolescents with hypercholesterolemia.

## Methods

Sixty-four children and adolescents (34 boys), aged 4.5 to 16 years, median age 9 years, were enrolled in an open-label two years’ prospective study. All children were recruited from the Lipid Outpatient Clinic of the 2^nd^ Department of Pediatrics of Athens University at “P. & A. Kyriakou” Children’s Hospital. The study was in accordance and approval by the Institutional Review Board of “P. & A. Kyriakou” Children’s Hospital. Written informed parental consent was obtained prior to participation.

Exclusion criteria included renal, hepatic or thyroid dysfunction, diabetes mellitus, chronic illness, long-term drug therapy and use of lipid-lowering drugs or dietary supplements known to affect cholesterol levels.

Children were assigned to two groups based on LDL-C levels which were verified more than once. Thirty children with LDL-C levels ≥3.4 mmol/l (130 mg/dl) comprised the hypercholesterolemic group and thirty four children with LDL-C levels <3.4 mmol/l the control group.

Hypercholesterolemic children were placed on a Step-II diet [8] according to the instructions of our Dietary Department. Six to 12 months later, a yogurt-drink, containing 2 g of plant sterols, replaced a dairy product with similar fat and carbohydrate content in their daily diet. Lifestyle and eating habits were maintained. Twenty five out of thirty children with hypercholesterolemia were re-examined within 6 to 12 months after the introduction of the sterol-enriched yogurt drink. Children who did not comply with daily sterol supplementation or dietary modifications, altered their pubertal status or BMI category or received other supplements known to affect cholesterol levels were considered dropouts. Finally, four children were excluded due to incompliance (compliance rate 86.6%) and one child due to shift in Tanner’s stage.

Height was measured to the nearest 0.1 cm for all the children, using a manual height board, weight was measured using an electronic scale to the nearest 0.1 kg and BMI was calculated using the standard formula kg/m^2^. International growth charts were used [10]. In addition, a standardized age- and sex-specific growth reference was used to calculate body mass index standard deviation scores (BMI-SDS).

Venous blood samples were collected in the morning (8.00-9.00 a.m.) after 12-hour overnight fasting. All participants were healthy at the time of blood sampling. TC, HDL-C and TGs levels were measured in serum samples using enzymatic methods (Roche Diagnostics, Cobas Integra 800). Apolipoprotein A-I (ApoA-I), ApoB, and Lp(a) levels were determined using an immunonephelometric assay (Siemens BNII Nephelometer Analyser). NonHDL-C was calculated as TC minus HDL-C. A direct homogenous assay was used to measure LDL-C levels (Roche Diagnostics, Cobas Integra 800). All values are expressed either in mmol/l or μmol/l. For the determination of sdLDL-C, samples were collected in EDTA tubes, were placed in ice and were immediately centrifuged at 4000 rpm (2000 × g) for 10 minutes at 4°C. The supernatant was isolated and stored at −80°C until further analysis. A commercially available kit (sLDL-EX “SEIKEN”, Randox Laboratories Ltd, UK) was used for the direct quantitative determination of sdLDL-C (Roche Diagnostics, Cobas Integra 800). Reference Intervals were investigated in adult population and the 75^th^ percentile value, being 0.9065 mmol/l (35 mg/dl), was selected as the cut-off point for CHD risk assessment. The linearity range of the assay was 0.1036-2.59 mmol/l (4.0 -100 mg/dl), Intra- Assay < 2%, Inter - Assay < 3% and Sensitivity 0.170-0.230 mg/dl [11]. Serum liver enzymes, creatinine, calcium, phosphorus, alkaline phosphatase, thyreotropin and thyroid hormones were evaluated in all children and re-evaluated in the hypercholesterolemic group within 6–12 months after the dietary intervention.

Statistical analysis was performed using STATA for Windows v8.5, (StataCorp, Texas, USA, 2006). Data are presented as mean +/−SD, median and range. Non-parametric methods were used (Wilcoxon paired test, Mann-Whitney *U* test and Kruskal-Wallis test, where applicable) to compare differences of numerical variables between the groups. Further correlations between the variables were made with the use of the non parametric Spearman’s rho statistic. P < 0.05 was considered as statistically significant.

## Results

The comparison of the body mass index standard deviation scores (BMI-SDS) between the control and the hypercholesterolemic group did not yield any statistically significant results (p = 0.547). Furthermore the BMI-SDS of the latter group did not change significantly after the introduction of the yogurt-drink (p = 0.621) (Table 1).

**Table 1:** Anthropometric measurements and lipid profile in the study population

	**Hypercholesterolemic group**		**P**	**Controls baseline**	P*	P**
	Before plant sterols*	After plant sterols**				
BMI	16.93 ± 2.64 16.65(13.1-21.6)	17.7 ± 2.89 17.1(13.2 ~ 22.4)		19.29 ± 3.08 18.8(13.1-27.8)		
BMI- SDS	0.29 ± 1.31 0.27(−1.79-2.51)	0.48 ± 1.34 0.68(−1.91 ~ 2.2)	0.62	0.77 ± 1.28 0.9(−2.2 ~ 3.85)	0.55	0.68
sdLDL-C	1.06 ± 0.23 1.04(0.71-1.59)	0.92 ± 0.25 0.84(0.44-1.47)	<0.001	0.60 ± 0.11 0.64(0.33-0.78)	<0.001	<0.001
TC	7.00 ± 1.45 6.90(5.13-11.42)	6.34 ± 1.57 5.92(3.83-9.95)	<0.001	4.50 ± 0.54 4.60(3.26-5.56)	<0.001	<0.001
LDL-C	5.20 ± 1.37 4.91(3.50-9.27)	4.55 ± 1.57 4.27(2.20-8.31)	<0.001	2.59 ± 0.36 2.64(1.76-3.34)	<0.001	<0.001
NonHDL-C	5.49 ± 1.50 5.40(3.39-10.13)	4.81 ± 1.66 4.37(2.49-8.96)	<0.001	2.87 ± 0.44 3.03(1.94-3.57)	<0.001	<0.001
HDL-C	1.53 ± 0.44 1.53(0.57-2.72)	1.53 ± 0.41 1.53(0.91-2.85)	0.58	1.66 ± 0.47 1.58(0.98-2.64)	0.38	0.31
TGs	0.73 ± 0.33 0.64(0.36-1.63)	0.65 ± 0.24 0.60(0.35-1.40)	0.28	0.68 ± 0.25 0.63(0.28-1.24)	0.68	0.74
ApoA-I	50.88 ± 9.60 51.25 (31.67-86.87)	54.45 ± 12.46 53.02 (40.21-80.43)	0.22	53.02 ± 10.32 53.02 (32.74-77.94)	0.48	0.82
ApoB	2.38 ± 0.58 2.33(1.65-4.20)	2.09 ± 0.53 1.98(1.27-3.60)	0.004	1.31 ± 0.24 1.41(0.76-1.67)	<0.001	<0.001
Lp(a)^§^	1.04 ± 0.91 0.77(0.08-3.11)	1.2 ± 0.72 0.97 (0.09-2.68)	0.91	0.60 ± 0.68 0.31(0.08-2.08)	0.03	0.002

Laboratory data are expressed as mean ± SD, median and range in mmol/l and in ^§^μmol/l.

*Abbreviations*: BMI: body mass index; BMI-SDS: body mass index standard deviation scores; sdLDL: small dense low density lipoprotein-cholesterol; TC: total cholesterol; LDL-C: low density lipoprotein-cholesterol; NonHDL-C: non high density lipoprotein-cholesterol; HDL-C: high density lipoprotein-cholesterol; TGs: triglycerides; ApoA-I: apolipoprotein A-I; ApoB: apolipoprotein B; Lp(a): lipoprotein (a).

*comparison between hypercholesterolemic group before plant sterols and controls baseline **comparison between hypercholesterolemic group after plant sterols and controls baseline.

**Figure 1:**
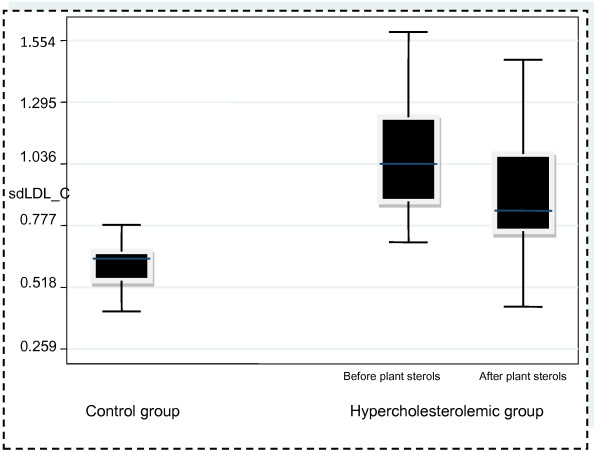
Distribution of sdLDL-C in controls and in hypercholesterolemic children.

Children and adolescents with hypercholesterolemia had statistically significant higher concentrations of sdLDL-C compared with controls (p < 0.001). The introduction of the sterol-enriched yogurt drink resulted in a significant decrease of sdLDL-C levels (p < 0.001), as well as TC, LDL-C, NonHDL-C (p < 0.001) and ApoB (p 0.004, Table 1). Nevertheless, sdLDL-C remained significantly higher compared with controls (p < 0.001, Figure 1). HDL-C, ApoA-I, Lp(a) and TGs levels were unaffected (Table 1).

LDL-C and sdLDL-C levels decreased by ≥10% in 16 out of 25 children (64%), while in 3 more children a decrease between 6% -9% was achieved. Moreover, the median decrease was 16.6% and 13% for sdLDL-C and LDL-C respectively. The percentage of decrease was independent of baseline LDL-C and sdLDL-C levels (p = 0.34). Additionally, no significant correlation was observed between sdLDL-C concentration and sex, age or BMI in both groups. Similarly, sdLDL-C did not correlate with HDL-C, TGs or Lp(a) levels (rho = 0.13 p = 0.31, rho = −0.07 p = 0.57 and rho = 0.13 P = 0.33 respectively). All other serum biochemical and hormonal markers remained within normal range.

## Discussion

In the present study we observed that hypercholesterolemic children had significantly higher sdLDL-C levels compared with controls. The distribution of sdLDL-C subclasses in children with primary dyslipidemia is yet to be assessed. In an adult study, patients with FH had decreased LDL peak particle diameter- i.e. the diameter of most abundant subclasses of LDL particles- compared with controls and an accumulation of midsize LDL molecules [12]. In another study, adults with familial combined hyperlipidemia (FCH) had significantly higher concentrations of ApoB and sdLDL compared with healthy individuals [13]. With respect to secondary dyslipidemia, 54% of obese adolescents included in a study were characterized by phenotype B [14]; according to existing literature only 2-13% of the healthy pediatric population is characterized by this phenotype [15-22]. In a later study, forty percent of obese children with lipid abnormalities were found to have sdLDL particles [23]. This predominance is even more profound in children and adolescents with diabetes mellitus, reaching 86.7%, whereas only 11% of controls belonged to phenotype B. Additionally, youths with diabetes had significantly smaller mean LDL diameter than controls [24].

Studies have shown that sdLDL particles are found over 3 times more often in healthy adults than in children. Individuals with this predominance exhibit a three-fold higher risk for myocardial infarction compared with those with phenotype A [5].

The present study demonstrated that the daily consumption of 2 g of plant sterols in children and adolescents with dyslipidemia significantly decreased sdLDL-C levels. To the best of our knowledge there are no previous reports of the effect of plant sterols on sdLDL-C in children. Previous data in adults was scarce and conflicting, partly due to multiple interventions placed concurrently, thus making it difficult to attribute the positive effect on LDL size solely to sterols [25,26]. A recent adult study in patients with metabolic syndrome reported a significant decrease in sdLDL-C levels after a two month supplementation with phytosterols [27]. This decrease in the sdLDL-C levels when a plant sterol supplementation diet was used, could be an additive benefit to the well documented lipid-lowering one. More studies in adult and pediatric population, at high risk of atherosclerosis, are needed in order to understand up to what extent and how plant sterols influence the phenotype of LDL particles. Interestingly, in our study, children with hypercholesterolemia presented significantly higher sdLDL-C levels in comparison to controls even after dietary modification, implying that simultaneous interventions might be necessary to adequately decrease atherogenic LDL particles.

No correlation was observed between sdLDL-C levels and BMI, age or sex in youths with or without lipid abnormalities. The literature concerning these parameters is controversial [16-22]. In the Bogalusa heart study, weight was negatively associated to LDL size and boys had smaller mean LDL particle size than girls [17]. A Canadian study of more than 2000 healthy youths found that the prevalence of phenotype B was only 2% and reached 7% in children with increased BMI, while no sex differences were observed [21]. Two other studies showed similar distribution of sdLDL particles in boys and girls, the latter study also found no correlation between LDL particle size and weight-for-height [16,22]. In our study the majority of children had normal BMI, which may explain the absence of correlation between BMI and sdLDL-C levels. Finally, research has not yet established a correlation between age and phenotype B in children [21].

There is sufficient data to support the lipid-lowering effect of phytosterols in patients with lipid abnormalities. Meta-analysis of clinical trials in adults has shown that daily intake of 2 g of phytosterols significantly decreases TC and LDL-C with no effect in the majority of studies on HDL and TGs levels [7]. In children, fewer studies have evaluated the effect of plant sterols on lipids. An approximate 10% decrease in LDL-C levels was observed in children with FH after daily consumption of 1.6 g of a plant sterols-enriched spread. TC and ApoB concentrations were also decreased to a significant degree, with no serious adverse effects [28]. Based on current data experts recommend the use of plant sterols only for children with hypercholesterolemia who do not achieve desired LDL-C levels after lifestyle changes [8].

The present study demonstrated that in hypercholesterolemic youths TC, LDL-C and NonHDL-C concentrations were significantly decreased within 6 to 12 months from the initiation of a diet supplemented with plant sterols. ApoB levels were also reduced to a smaller degree. LDL-C and NonHDL-C levels were reduced more than 10% in almost two thirds of the children studied, while the median reduction was approximately 13%. The percentage reduction in lipid concentrations was independent of the baseline levels. Plant sterols supplementation had no effect on HDL-C, TGs and Lp(a) levels. The yogurt-drink was well tolerated with no side effects. The main limitation of our study was the small number of participants, necessitated by cost constraints for laboratory measurements.

## Conclusions

In conclusion, in our population of hyperlipidemic children and adolescents, plant sterol supplementation had a beneficial effect not only on the levels of total LDL-C but also on its more atherogenic small, dense particles. It seems to be a safe and efficacious approach in an attempt to lower the cholesterol levels in children with hyperlipidemia at high risk of early atherosclerotic disease, thus postponing the initiation of medical treatment. In addition, plant sterols do not decrease HDL-C concentration and therefore their use can be a tempting first line choice along with other dietary modifications for hyperlipidemic children and adolescents. Finally, more studies in pediatric populations at high risk of atherosclerosis are needed in order to reinforce the significance of our findings and elucidate the role that plant sterols play in the phenotype of LDL particles.

## Abbreviations

sdLDL-C: Small dense low density lipoprotein-cholesterol; TC: Total cholesterol; LDL-C: Low density lipoprotein-cholesterol; NonHDL-C: Non high density lipoprotein-cholesterol; ApoB: Apolipoprotein B; BMI: Body mass index; HDL-C: High density lipoprotein-cholesterol; Lp(a): Lipoprotein a; TGs: Triglycerides; sdLDL: Small dense LDL; lbLDL: Large buoyant LDL; CHD: Coronary heart disease; FH: Familial hypercholesterolemia; BMI-SDS: Body mass index standard deviation scores; ApoA-I: Apolipoprotein A-I; FCH: Familial combined hypercholesterolemia.

## Competing interests

The authors declare that they have no competing interests.

## Authors’ contributions

AG and SV contributed to the design of the study, the enrolment of the participants and the interpretation of data. DG, AM and AD designed and performed the laboratory measurements. AG and CT conducted the statistical analysis. AG, SV, AS and LK participated in the writing of this manuscript. All authors read and approved the final manuscript.
